# Age and Sex Differences in Physical Activity of Portuguese Adults and Older Adults

**DOI:** 10.3390/healthcare11233019

**Published:** 2023-11-22

**Authors:** Teresa Bento, Maria P. Mota, Anabela Vitorino, Diogo Monteiro, Luís Cid, Nuno Couto

**Affiliations:** 1 Sport Sciences School of Rio Maior, Polytechnic of Santarém (ESDRM-IPSantarém), 2040-413 Rio Maior, Portugal; anabelav@esdrm.ipsantarem.pt (A.V.); luiscid@esdrm.ipsantarem.pt (L.C.); ncouto@esdrm.ipsantarem.pt (N.C.); 2 Research Center in Sports Sciences, Health Sciences and Human Development (CIDESD), 5001-801 Vila Real, Portugal; mpmota@utad.pt (M.P.M.); diogo.monteiro@ipleiria.pt (D.M.); 3 Department of Sport of Science Exercise and Health, School of Life and Environmental Sciences, (ECVA), University of Trás-os-Montes and Alto Douro (UTAD), 5000-801 Vila Real, Portugal; 4 School of Education and Social Sciences (ESECS), Polytechnic of Leiria, 2411-901 Leiria, Portugal

**Keywords:** accelerometry, physical activity, public health, recommendations, ageing

## Abstract

This study aims to explore data on objectively measured physical activity from a sample of adults and older adults and to investigate the achievement of the minimum physical activity required for health benefits. Participants, 257 women (age 58.99 ± 18.93 years, BMI 26.75 ± 4.57 kg·m^−2^) and 178 men (age 49.22 ± 20.39 years, BMI 26.81 ± 3.51 kg·m^−2^), wore an accelerometer for 4 to 7 days. Approximately 40% of the time was spent daily in sedentary behaviors during waking hours. Our results do not sustain the suggestion that adult men are more physically active than women. The results indicated a decrease in intensity of physical activity with age, in men and women, but not in successive age groups. Around 75% of adults and 30% of older adults accumulated the minimum daily physical activity for health benefits, in periods shorter than 10 min, above moderate intensity. The number of steps taken per day revealed that most men and women (except the oldest) attained or exceeded the lower threshold for deriving health benefits. To obtain relevant health benefits, future physical activity interventions should aim at reversing the amount of sedentary behaviors, emphasizing increments in, at least, the lower levels of PA, and maintaining walking habits.

## 1. Introduction

Recent research based on systematic reviews and meta-analyses (supported mostly by epidemiological studies consisting of large cohorts) has revealed a consistent connection between physical activity (PA) and a reduced risk of premature mortality and the prevention of various chronic diseases [1,2,3,4,5,6,7].

Based on the existing scientific evidence, guidelines for minimum PA an individual should engage in to obtain health benefits have been stated and updated. Recommendations for PA from the World Health Organization (WHO) in 2010 [8] suggested that adults aged 18 to 64 years should accumulate at least 150 min of moderate-intensity aerobic PA throughout the week or at least 75 min of vigorous-intensity aerobic PA throughout the week, or an equivalent combination of both. That aerobic activity should be performed in bouts of at least 10 min duration, and for additional health benefits, adults should increase time spent in higher intensities and include muscle-strengthening activities throughout the week [8]. The concept of accumulation involved achieving the 150 min weekly goal by engaging in activities in multiple shorter bouts, each lasting at least 10 min, spread throughout the week, and then adding together the time spent during each of these bouts. For example, one could reach the goal by completing 30 min bouts of moderate-intensity activity five times per week.

Later, updated scientific evidence prepared for the “2018 US Physical Activity Guidelines Advisory Committee Scientific Report to the Secretary of Health and Human Services” [3] led to an update of the WHO’s recommendations for PA in 2020; for the first time, information was provided on the associations between sedentary behavior and health outcomes [9].

Both organizations have reasserted their prior guidelines, emphasizing that adults should engage in aerobic PA for substantial health benefits. This activity should consist of at least 150 to 300 min per week of moderate-intensity exercise or 75 to 150 min per week of vigorous-intensity exercise, or a combination of both. Ideally, individuals should distribute their aerobic activity throughout the week. Considering that relevant research reported that the average time per day accumulated in frequent shorter bouts of PA of moderate intensity has similar effects to those observed when the average time per day is accumulated in bouts of moderate intensity that last at least 10 min in duration [2,10], the previous recommendation specifying that PA should be accumulated in bouts of at least 10 min for optimal health outcomes has been eliminated due to insufficient scientific support [10,11]. The minimum bout duration of PA for positive health benefits remains unclear [11], but such a recommendation may facilitate individuals in adhering to the recommended levels of PA [11,12]. Regardless, several studies using accelerometers continued to include the 10 min bouts in their analyses [13,14,15,16].

The number of steps taken per day (especially the goal of 10,000 steps per day) is widely promoted as optimal for health. While the official PA recommendations do not specify a daily step count due to the limited scientific evidence available to determine the precise number of steps required for maintaining health, it is noteworthy that daily step counts have proven effective as a goal for adults to attain the recommended levels of PA [17,18,19].

The reference for taking 10,000 steps per day [20] has been widely adopted in research in the last 10 years. However, a recent systematic review [11] found consistent evidence from longitudinal data that walking an additional 1000 steps per day can help lower the risk of all-cause mortality and cardiovascular disease (CVD) morbidity and mortality in adults, and that health benefits are present below 10,000 steps per day. The study by Jefferis [21] specifically indicated a 15% reduction in mortality associated with an increase of 1000 steps/day. Also, the meta-analysis by Paluch et al. [22] confirmed that mortality benefits, particularly for older adults, can be seen at levels below this threshold, which may have implications for forthcoming public health PA guidelines. More research is needed to identify the number of steps per day that should be recommended; however, data from the literature synthesis demonstrate the health potential of everyday walking for large segments of populations [22,23].

Despite the vast evidence showing the health benefits of PA, studies [4,7,13,15,16,24,25] demonstrate that, in general, adults and older adults do not engage in enough PA to have a positive impact on their health, as suggested by the PA recommendations for health [4,24,26].

These data become meaningful, as a decline in PA is associated with an increase in age. Several epidemiological studies showed the general decline in PA with advanced age past adulthood, in consequence of the decline in strength and physical function, increased chronic disease prevalence and was related to psychosocial factors (including life transitions such as retirement) [9,27,28] and to other social constraints preventing participation in PA programs [29]. These studies also suggest that men are more active than females at older ages and that these differences may be attributable to diurnal patterns of PA.

Research on sex differences revealed that women self-report less PA than men [9,30,31,32]. However, in studies that used more objective measures, such as motion sensors, the findings diverge [31,33,34].

Historically, assessments of PA levels and compliance with guidelines have primarily relied on self-reported measures like questionnaires, often leading to an overestimation of activity levels. An increasing number of published reports have used objective measures to assess PA; however, these studies are predominantly performed on cross-sectional populations or on children or youth groups from the United States, the United Kingdom, and Japan [35]. There is less published research using samples of adults and older adults from other regions, especially Europe [13,16,25,35]. Therefore, valuable information from different countries and age groups, is of major importance to identify how levels of PA are achieved and how to promote more adequate intervention strategies according to population characteristics and behaviors.

Therefore, this study aims to explore data on objectively measured PA from a sample of adults and older adults to establish comparisons between age and sex groups as well as to investigate the achievement of the minimum PA required for health benefits.

## 2. Materials and Methods

This was a descriptive, cross-sectional study that aimed to explore data on objectively measured physical activity from a sample of adults and older adults and to investigate the achievement of the minimum physical activity required for health benefits.

### 2.1. Participants

Eligible participants were physically healthy male and female adults above 20 years of age, willing to wear an accelerometer every day, and residents within the geographic area defined for the study (i.e., Municipality of Vila Real). Participants were recruited by word of mouth and selected based on a non-probabilistic convenience sample, based on the criterion of recruitment by accessibility and availability.

Of the 625 subjects initially recruited and evaluated, 190 did not wear the accelerometer for at least 4 days, had battery failure, or had equipment malfunction, and for these reasons the final sample included 257 women (20 to 96 years old, BMI 26.75 ± 4.57 kg·m^−2^) and 178 men (20 to 88 years old, BMI 26.81 ± 3.51 kg·m^−2^). Considering the total population in Vila Real (*n* = 36,994), a 95% confidence interval, and a 5% margin of error, the total sample size to be considered representative is 381, which is respected in this study.

To allow for the comparison of data, participants were classified into three categories based on their ages (20–39 years, 40–59 years, and ≥60 years) according to stratification data from other studies [36,37].

### 2.2. Procedures

The study design and experimental procedures were explained to potential participants. After recruitment, participants willing to participate signed an informed consent and completed a socio-demographic questionnaire in addition to being assessed for height and weight. Body Mass Index (BMI), used solely for describing the characteristics of the sample, was calculated by applying the following formula: (weight (kg)/height (m)2), and expressed as (kg·m^−2^).

Subjects were fitted for a belt with an attached accelerometer fitted closely around their waist [38,39] and were instructed to wear the accelerometer during all waking hours for four to seven consecutive days, except when showering, bathing, or swimming. Participants were asked to annotate the time on a brief log.

The time frame for data collection was the entire year, except for the months of August, as most participants were on vacation and refused to wear the device.

### 2.3. Accelerometer, Data Reduction, and Outcome Measures

The ActiGraph models (ActiGraph GT1M, ActiGraph, Pensacola, FL, USA) were used to objectively assess PA and were calibrated according to the manufacturer’s instructions. The validity and reliability of accelerometers within and across monitors have been previously tested [40].

Freedson cut-off points [41], adapted by Matthews and colleagues [33], were used to evaluate the time spent in activities of different intensity by adults (under 60 years old). The intensity categories of PA were defined as the following: inactive (from 0 to 500 ct·min^−1^), light (500 to 1952 ct·min^−1^), and moderate to vigorous ((MVPA) (above 1952 to 5724 ct·min^−1^)). These thresholds were chosen because they derived from an adult population and were calibrated for walking, the most globally performed activity.

Considering that cut-off points for older adults (above 60 years old) are not validated, and the use of a single cut-point for all adults may lead to an underestimation of moderate PA intensity in the elderly, cut-off points by Davis [25] were used. For the older group, categories were defined as the following: sedentary activity (less than 200 ct·min^−1^), light activity (less than 3 METS, ranging from 200 to 1999 ct·min^−1^), and MVPA (more than 3 METS, above 1999 ct·min^−1^). These cut-off points were chosen because they were previously used in other studies with similar samples [25,42,43,44].

Intensity categories defined for adults and older adults corresponded to the same metabolic equivalents but were given different designations by the researchers (e.g., inactive for adults and sedentary for older adults). The categories of sedentary activity, light activity, and MVPA were used for both age groups to standardize the terminology and to facilitate data presentation, interpretation, and comparison.

The data used for analysis consisted of at least 4 days of data collection for a minimum of 10 h per day, in cycles of 1 min; 60 min or more of consecutive zero counts were considered missing data or non-wearing time and were eliminated [45].

To analyze the achievement of the minimum PA required for health benefits, the guidelines by the WHO [9] were considered. Bearing in mind the possibility to accumulate minutes of daily PA to achieve the 150 min weekly goal, preconized by the guidelines, adherence to the recommendations for PA was analyzed if participants achieved 30 min of PA throughout the day or in bouts of more than 10 min above the MVPA threshold. While current recommendations no longer incorporate the mention of PA bouts exceeding the MVPA threshold as a means to attain health benefits because this relationship is still unclear [11,12], this was the messaging in the context of public health at the time of collection of data. This inclusion also enables the comparison of data with other studies that did consider 10 min bouts and the 30 min daily reference in their analyses [13,14,15,16].

Given the lack of evidence regarding the specific daily step count recommendation for health, it is important to acknowledge the health benefits associated with regular walking for significant portions of the population [22,23]. In light of this, an examination of the widely adopted guideline of aiming for 10,000 steps per day, as proposed by Tudor-Locke and Basset [20], was also conducted.

Our outcome variables were the following:

(a) Daily time spent in different intensity activities (min·day^−1^);
(b) Average intensity or total volume of PA (ct·min^−1^) per day;
(c) Steps per day (steps/day).

Data were reduced using MAHUffe software, available from www.mrc-epid.cam.ac.uk/ (accessed on 1 March 2023).

### 2.4. Statistical Analysis

Statistical analysis was conducted using PASW Statistics version 18 (SPSS Inc, Chicago, IL, USA) and Excel 2011 (Microsoft Corporation, Redmond, WA, USA). Descriptive statistics were expressed as absolute and relative frequencies, means, and standard deviations. Non-parametric statistics were preferred due to the skewed distribution of most variables. Comparisons between sex were made using Mann–Whitney‘s U test. Comparisons between age groups were made using the Kruskal–Wallis test. Significant differences were identified using Mann–Whitney’s U (significance level was set at *p* ≤ 0.016). Spearman’s Rho was used to check correlations between variables (statistical significance level was set at *p* ≤ 0.05).

## 3. Results

The studied population consisted of 435 subjects (59% women); sample characteristics are summarized in Table 1. The final sample included 69.4% of the eligible sample of 627 participants.

The average number of valid days the accelerometer was worn for the whole sample was 4.98 days, and the time worn ranged from 10.22 to 18.48 h per day.

Time spent performing different intensity activities, daily average (ct·min^−1^), and steps taken (mean ± SD), categorized by sex and age groups, are presented in Table 2.

Results of comparison between age and sex groups will be further presented considering the subtopics: sedentary time, total PA, daily time spent in different intensities activities, and minimum PA for health benefits.

Sedentary time, total PA, and daily time spent in different intensity activities: Sex differences in daily average ct·min^−1^ were only found between groups ≥ 60 years old (*p* = 0.047), with men attaining higher amounts of average daily PA. Men accumulated 62 min (mean values) more in sedentary time in the 40–59 age group (*p* < 0.001) and spent 14 more min (mean values) in MVPA above 60 years (*p* = 0.03) than women.

The number of steps taken per day differed between sexes in the youngest (U = 0.012) and oldest groups (*p* = 0.03).

When considering differences between age groups, women between 20 and 39 years old spend less time in light activity than women 40 to 59 years old (U = 1420; *p* = 0.009). Significantly higher values of MVPA, daily average ct·min^−1^, and steps were observed in the younger females compared to older ones (≥60 years) (U = 1088, U = 1335, and U = 1284, respectively; all *p* < 0.001). The values observed in younger women for these variables are two times higher than the values observed in older women. From the 40 to 59-year-old and ≥ 60-year-old age categories of females, there was a decrease in light activity, MVPA, daily average ct·min^−1^, and steps taken (U = 3460.5, U = 2288.5, U = 2520, and U = 2284, respectively; all *p* = 0.000) but also an increase in time spent in sedentary activity (U = 3266.5, *p* < 0.001).

Comparison between the 20 to 39-year-old and the ≥ 60-year-old men also resulted in higher values observed in younger group in MVPA and daily average ct·min^−1^ but not in the number of steps taken (U = 1475.5, *p* = 0.001; U = 1567.5, *p* = 0.003; and U = 1634.5, *p* = 0.008, respectively). Men in the 40 to 59-year-old group spent significantly less time in MVPA compared to men in the ≥60-year-old age category (U = 901.5, *p* = 0.003).

Minimum PA for health benefits: When analyzing MVPA in detail, 72.3% and 66.3% of women between 20–39 years of age and 40–59 years of age, respectively, accumulated 30 min above MVPA per day. Approximately 30% of women in the older age category achieved the same number of minutes above this threshold. The results were quite similar for men, as 75% of 20–39-year-old men, 72.7% of 40–59-year-old men, and 38.7% of ≥60-year-old men accumulated 30 min per day above MVPA.

If sessions of 10 or more consecutive minutes of MVPA were considered to equal the recommendations of PA from the WHO [8], the percentage of non-compliers drops to 23% and 14% in females and males, respectively.

Finally, in our analysis of the number of steps in relation to the minimum 10,000 steps/day to be considered “active” [20], none of the sex and age groups achieved this recommendation in terms of mean values.

## 4. Discussion

Sedentary time, total PA, and daily time spent in different intensity activities: According to our data, approximately 40% of the time was spent in very low intensity behaviors during waking hours across all age and sex groups. These results are well in line with previous studies that report a high percentage of time spent in sedentary behavior [2,13,14,15,16,46,47]. Findings from a substantial sample encompassing England, Portugal, Norway, and Sweden [13] revealed that, on average, participants accumulated approximately 8 to 9 h of sedentary time per day. Notably, 80% of these participants accrued at least 7.5 h of sedentary time daily. These figures closely resembled the results from the 2005–2006 National Health and Nutrition Examination Survey (NHANES) accelerometer study in the USA, which reported an average of 8 h of sedentary time per day [46].

Comparing data of the time spent in sedentary behavior with other research involving European samples [13,15], we registered substantial higher values than other studies. This may be due to the fact that those authors used a lower cut-off value for sedentary activity (<100 cts·min^−1^) than the one we used in our study (<500 cts·min^−1^). Our results were based on a more conservative limit for sedentary behavior, which may account for more time spent in sedentary activities. If the same cut-off point was applied, potentially, less time would be considered sedentary time and the time would instead account for light PA, suggesting that our sample would be less sedentary compared to others. This is relevant when comparing the present results with the study by Batista [15] of a representative sample of the Portuguese population; without considering this information, one could conclude that our participants spend twice the time in sedentary behavior, which may not correspond to reality. The reason these thresholds were chosen was mainly because they derived from an adult population and were calibrated for walking, the most globally performed activity. Also, differences in the age categorization must be noted, as our 20–49 age group corresponds to two age groups (1–29 and 30–39) in Batista´s study [15], making it more difficult to compare results. Differences in definitions of activity intensity and analytical techniques can introduce bias when comparing studies involving objective PA data, potentially leading to inconsistent results, even when working with the same dataset.

Regarding sedentary behavior, data from a recent systematic review and harmonized meta-analysis indicates that a higher risk of death was observed from 9.5 or more hours per day of sedentary time [2], which indicates a concern for the time spent in sedentary behavior by our sample.

No differences between sexes were observed, except for men in the 40–59 age group accumulated 62 min (mean values) more sedentary time than women of the same age group.

Regarding older people, our results agree with previous studies that show that approximately 60% of their time, corresponding to more than to 8 h per day, is spent in sedentary behaviors [14,16,48]. According to Jefferis [21], for older men, a 30 min daily increase in sedentary behavior corresponded to a 15% higher risk of mortality. This suggests that, among older men, even lighter-intensity activity can play a significant role in mortality prevention.

Unexpectedly, our data do not confirm previous findings that sedentary activity increases with age [13,15,16,45]. In our study, except for 40–59 to ≥60-year-old women, where an increase in time spent in sedentary activity was observed, these behaviors tended to be stable. Explanations for this may be associated with the fact that the chosen cut-off points for sedentary behavior and light PA may account for more time spent in sedentary activities than in lower-level PA, and therefore, changes in sedentary behavior are difficult to establish.

With regard to PA across age groups, our results from the daily average ct·min^−1^ between sexes contradict earlier reports suggesting that adult men are more physically active than women [15,38,49]. However, detailed analysis of the intensities of PA that led to the same totals of PA (in daily average ct·min^−1^) in men and women revealed differences between sexes. Accounting greatly for adult men´s total PA is the time spent in vigorous PA, which is barely represented in adult women´s total PA. As observed in previous studies, women spend more time in light PA [13,14,16], where activities that are normally not classified as real exercise, such as walking and household and child care activities, are included [16]. For this matter, it is important to refer the results from a recent systematic review and meta-analysis [2] that indicate that higher risk reductions in mortality can be seen at lower intensity levels of PA.

Regarding older adults’ daily average ct·min^−1^, our results align with previous research, showing distinctions in the older age group, with men attaining higher amounts of average daily PA than women [15,16,25].

Analyzing MVPA in detail, no differences were found between men and women, until above 60 years old, when males attained higher values, as previously reported [15,16,25,44].

Across age groups, women above 60 years of age compared to those 40 to 59 years old showed a decrease in all levels of PA and in steps taken per day, while in men, the only significant reduction was in time spent in MVPA between the same age categories. These results have been stated earlier by other researchers [13,16,25]. Results from our study also imply a tendency to decrease the intensity of activities with age, which may relate to the decline in physical function linked to age as well as the reduction in physical activity related to transportation and work, as retirement may take place [14,15,16].

Previous studies have shown a decline in PA with age [13,16], and in our study, younger adults were in fact more physically active than their elders; however, it was not observed that PA is lower in successive age groups, as was also seen by Batista [15]. This might be explained by the fact that the age-related decline in PA is most prominent in the transitions from youth to adulthood and from adulthood to retirement age [16].

### Minimum PA for Health Benefits

Adherence to the recommendations for PA throughout the day [9] revealed that 72.3% and 66.3% of women 20–39 years of age and 40–59 years of age, respectively, were compliant and that 75% of 20–39-year-old men and 72.7% of 40–59-year-old men achieved the recommendation, confirming results from previous studies in Europe [15]. In the study by Loyen [13], participants reached a lower percentage of compliance (32%); however, compliance was defined as accumulating 150 min/week based on total time in MVPA [24], which is somewhat different from our definition of 30 min of at least moderate intensity PA per day [9].

Men and women above 60 years old accumulated higher values than previous studies developed with similar samples from European countries using the same cut-off points [25,42]. Our results also indicate that 30% of women and 38.7% of men in the older age category were compliant with the guidelines, reaffirming recent studies´ results [15] and confirming that in this age group these guidelines may be hard to follow [14]. As stated previously, interventions that aim at increasing levels of total PA (at any intensity level) and decreasing the amount of time spent in sedentary behaviors may be associated with a significant reduced risk in premature death [2] and may lead to better adherence for previously sedentary persons [12].

The percentage of adults complying to the recommendations decreased to 14% in men and 23% in women when bouts of 10 or more consecutive minutes above MVPA [8] was considered. Previous studies with adult populations found similar results, from 20% to 32% [13,16]. A substantial difference was observed in the 2003–2004 NHANES accelerometer study from the USA, which reported that approximately 97% of the participants did not meet the PA recommendations when considering similar 10 min MVPA bouts [46]. While recent revisions to both national and international PA guidelines have eliminated the requirement for PA to be accumulated in bouts of at least 10 min, the minimum bout duration of PA for positive health benefits remains unclear [11]. However, relevant research reported that the average time per day accumulated in frequent shorter bouts of PA of moderate intensity has similar effects to those observed when average time per day is accumulated in bouts of moderate intensity that are at least 10 min in duration [2,10]. Also, one recent systematic review suggested that PA accumulated in many patterns of bout duration, intensity, or daily/weekly frequency is associated with a range of beneficial health outcomes in adults [11], backing up the updated guidelines.

In light of this and research worldwide, where individuals across all age and sex groups struggle to comply with such a guideline, the new recommendations [9] have the potential to increase individuals’ adherence to the prescribed levels of PA as well as to enhance the adoption and maintenance of regular PA for previously sedentary persons. In another perspective, considering that time constraints are a commonly mentioned obstacle to PA, a suggestion that permits individuals to engage in brief activity sessions at different times during the day, as opposed to requiring them to allocate a continuous time block in their schedules, is naturally appealing.

Analysis of the number of steps taken per day revealed that none of our age or sex groups accomplished the minimum 10,000 steps/day to be considered “active” [20]. In women, the number of steps taken per day differed between the youngest and oldest group, while men showed no difference across age groups. Differences between sex were observed in the age groups of 20–39 years and above 60 years old, with women accumulating more steps per day in the youngest category and men in the oldest.

By merging the data indicating that women in the 20–39 age group take more steps and the previously analyzed information that adult women engage in lighter PA than men, it reinforces the notion that adult women participate in activities typically not classified as formal exercise, like walking, household chores, and childcare. Therefore, the widely accepted belief that men are more physically active than women, which is not applicable in our sample, might no longer hold true, as also was observed in Scandinavia [16].

Beyond the age of 60, especially for women, daily step counts decrease, which is consistent with earlier studies [43] and meta-analyses [50].

Recent research findings have indicated a progressively reduced risk of mortality among adults younger than 60 years old who achieve approximately 8000–10,000 steps per day and among adults aged 60 and older who reach around 6000–8000 steps per day [22]. When comparing these data with our sample, it becomes evident that both adult men and women in various age groups exceeded the lower threshold (at least) for deriving health benefits. Among the older age groups, women were the only group that did not attain the minimum, although the mean average came very close. These findings from our sample provide encouraging evidence of substantial PA levels.

While official PA guidelines refrain from specifying a daily step count due to the limited scientific evidence available for determining the exact number of steps essential for maintaining health, the concept of aiming for 10,000 steps per day, as proposed by Tudor-Locke and Basset [20], has gained widespread acceptance in research. It is worth noting that daily step counts have proven to be an effective target for adults to reach recommended levels of PA [17,18,19]. Furthermore, a recent systematic review by Brady [11] found consistent evidence from longitudinal data, indicating that adding an extra 1000 steps per day can significantly reduce the risk of all-cause mortality and cardiovascular disease morbidity and mortality in adults. Brady’s work [11] also highlights that health benefits can be attained below the 10,000 steps per day threshold, which our sample, in general, achieved in terms of steps taken per day. While further research is required to determine the specific daily step count to recommend, the available data from the literature syntheses underscore the health advantages of regular walking for substantial portions of the population [22,23].

In this study, PA data were objectively measured using accelerometers, which tends to be more reliable in the estimation of sedentary time and time spent in different levels of PA than subjective methods (such as questionnaires). Not without its challenges, the use of accelerometers depends on the correct use of the equipment for several days, the commitment of the participant, and proper functioning of the device itself. There was a significant loss of data because of insufficient wearing time or malfunctioning of the device (e.g., battery failure or equipment malfunction).

The cut-off points used in the present study to define PA levels derived from an adult population and were calibrated for walking, the most globally performed activity, and specific cut-offs were used to analyze data from the older age groups, to avoid an underestimation of moderate PA intensity in the older age group. In this sense, regarding data from sedentary behavior and time spent in light PA, our results were based on a more conservative limit for sedentary behavior (higher than other researchers), which may account for more time spent in sedentary activities. If the same cut-off point was applied, potentially, less time would be considered sedentary time and more would account for light PA, suggesting that our sample would be less sedentary compared to others.

Finally, this study was performed in a convenient sample of adults and older adults that lived in the defined study region; thus, these results may be specific to this geographic area. Therefore, other studies should be replicated in another region to account for these differences.

## 5. Conclusions

According to our data, a great amount of time was spent daily in very low intensity behaviors during waking hours across all age and sex groups.

Regarding daily average ct·min^−1^, our results do not sustain the suggestion that adult men are more physically active than women; however, detailed analysis of the intensities of PA that amounted to the same totals of PA confirm that adult women spend more time in light PA than men.

The results indicated a trend of decreased intensity of PA with age, in men and women; however, this was not observed in successive age groups.

Adherence to the recommendations of accumulating PA throughout the day was high in all sex and age groups, except for the older groups, where the percentage was lower for both sexes. Detailed analysis of MVPA revealed that it was accumulated in periods shorter than 10 min, especially in the older age groups.

The number of steps taken per day revealed that, except for women above 60 years, both age and sex groups attained or exceeded the lower threshold for deriving health benefits.

Based on the available research and data collected in this study, in order to obtain relevant health benefits, future PA interventions in this population should aim at reversing the amount of sedentary behaviors, emphasizing increases in, at least, the lower levels of PA, and maintaining walking habits.

## Figures and Tables

**Table 1:** Descriptive characteristics (age (years), weight (kg), height (cm), and BMI (kg·m^−2^)) of the subjects in total sample, sorted by sex.

		Total		20–39		40–59		>60	
	Total (*n* = 435)	Women (*n* = 257)	Men (*n* = 178)	Women (*n* = 47)	Men (*n* = 72)	Women (*n* = 84)	Men (*n* = 40)	Women (*n* = 126)	Men (*n* = 66)
	Mean ± SD	Mean ± SD	Mean ± SD	Mean ± SD	Mean ± SD	Mean ± SD	Mean ± SD	Mean ± SD	Mean ± SD
Age (y)	54.99 ± 20.10	58.99 ± 18.93	49.22 ± 20.39	28.96 ± 6.33	29.08 ± 5.65	52.90 ± 5.53	48.60 ± 5.15	74.13 ± 10.19	73.74 ± 7.86
Weight (kg)	69.95 ± 13.31	64.42 ± 11.22	77.93 ± 11.99	60.81 ± 10.10	81.73 ± 12.16	66.72 ± 9.52	77.71 ± 11.89	64.23 ± 12.30	73.82 ± 10.61
Height (cm)	161.43 ± 10.33	155.25 ± 6.96	170.33 ± 7.51	160.68 ± 0.05	175.00 ± 0.05	156.39 ± 0.05	170.30 ± 0.06	152.49 ± 0.06	164.84 ± 0.06
BMI (kg·m^−2^)	26.77 ± 4.16	26.75 ± 4.57	26.81 ± 3.51	23.49 ± 3.58	26.65 ± 3.65	27.31 ± 3.90	26.75 ± 3.68	27.58 ± 4.79	27.13 ± 3.26

Note. BMI—body mass index; SD—standard deviation.

**Table 2:** Time spent in different intensity activities (min·d^−1^), daily average (ct·min^−1^), and steps taken (mean ± SD), sorted by sex and by age group.

		Women				Men			
	Total	Total	20–39	40–59	>60	Total	20–39	40–59	>60
	(*n* = 435)	(*n* = 257)	(*n* = 47)	(*n* = 83)	(*n* = 127)	(*n* = 178)	(*n* = 72)	(*n* = 44)	(*n* = 62)
Sedentary (min·d^−1^)	1163.46 ± 103.27	1154.59 ± 110.51	1149.31 ± 90.06	1114.79 ± 102.81 ^(m) (b)^	1182.55 ± 114.58 ^(c)^	1176.28 ± 90.59	1183.76 ± 83.69 ^(l)^	1176.76 ± 76.03 ^(m)^	1167.25 ± 106.93
Light (min·d^−1^)	155.60 ± 76.97	163.76 ± 83.30	164.67 ± 61.81 ^(a)^	191.35 ± 65.00 ^(m) (d) (b)^	145.39 ± 95.46 ^(c)^	143.81 ± 65.23	145.12 ± 51.67	136.84 ± 45.01 ^(m)^	147.25 ± 88.09
MVPA (min·d^−1^)	40.28 ± 32.29	37.26 ± 31.73	54.51 ± 30.29 ^(b)^	49.31 ± 29.98 ^(b)^	23.01 ± 26.72 ^(n) (d) (c)^	44.64 ± 32.68	48.69 ± 27.40 ^(f)^	48.99 ± 29.40 ^(h)^	36.84 ± 39.00 ^(n) (i) (j)^
Daily average (ct·min·d^−1^)	323.94 ± 187.78	314.07 ± 192.98	411.28 ± 178.46 ^(b)^	390.14 ± 170.13 ^(b)^	228.38 ± 174.52 ^(o) (e) (c)^	338.18 ± 179.59	364.06 ± 143.47 ^(g)^	346.53 ± 152.18	302.22 ± 226.17 ^(o) (k)^
Steps	7772.64 ± 4299.71	7645.45 ± 4483.43	9683.49 ± 3459.20 ^(l) (b)^	9756.61 ± 3708.12 ^(b)^	5511.48 ± 4295.13 ^(p) (e) (c)^	7957.31 ± 4023.37	8251.02 ± 3176.24 ^(l)^	8598.75 ± 3535.46	7165.75 ±^(p)^ 5028.20

(a) Significant differences between women 40–59, *p* < 0.001; (b) Significant differences between women > 60, *p* = 0.009; (c) Significant differences between women 40–59, *p* = 0.009; (d) Significant differences between women 20–39, *p* < 0.001; (e) Significant differences between women 20–39, *p* = 0.009; (f) Significant differences between men > 60, *p* = 0.001; (g) Significant differences between men > 60, *p* = 0.008; (h) Significant differences between men > 60, *p* = 0.003; (i) Significant differences between men 20–39, *p* = 0.001; (j) Significant differences between men 40–59, *p* = 0.003; (k) Significant differences between men 20–39, *p* = 0.008; (l) Significant differences between men and women, *p* = 0.012; (m) Significant differences between men and women, *p* < 0.001; (n) Significant differences between men and women, *p* = 0.034; (o) Significant differences between men and women, *p* = 0.047; (p) Significant differences between men and women, *p* = 0.036.

## Data Availability

Data are available upon request to the first author.
